# New Challenges: Developing Gendered and Equitable Responses to Involuntary Exposures to Electronic Nicotine Delivery Systems (ENDS) and Cannabis Vaping

**DOI:** 10.3390/ijerph15102097

**Published:** 2018-09-25

**Authors:** Natalie Hemsing, Lorraine Greaves

**Affiliations:** Centre of Excellence for Women’s Health, Vancouver, BC V6H 3N1, Canada; lgreaves@cw.bc.ca

**Keywords:** electronic nicotine delivery systems (ENDSs), cannabis, vaping, involuntary exposure, sex, gender

## Abstract

Recreational cannabis use is in the process of being legalized in Canada, and new products and devices for both nicotine and cannabis vaping are being introduced. Yet, research on the harms of involuntary exposure to electronic nicotine delivery systems (ENDSs) and cannabis vaping is in its infancy, and there is a lack of investigation on sex-specific health effects and gendered patterns of exposure and use. We argue that responses to ENDS and cannabis vaping exposures should align with policy and progress on restricting exposure to tobacco secondhand smoke (SHS). Furthermore, we argue that sex, gender, and equity considerations should be integrated in both research and policy to benefit all Canadians.

## 1. Background

Progress on reducing secondhand smoke (SHS) exposure in Canada is facing new challenges. National data from Canada reveal that 16.1% of males and 10.5% of females have tried electronic cigarettes, and 14.9% of males and 9.7% of females have reported cannabis use in the past year [1]. Of those who reported cannabis use in the past year, 28% reported using a vaporizer to consume cannabis [2]. With the introduction and expansion of electronic nicotine delivery systems (ENDSs), combined with the legalization of recreational cannabis use in Canada, there is a need to consider the health effects of involuntary SHS exposure to these substances and review the existing policy and public health responses to SHS messaging and smoke-free legislation. In the following commentary we argue that in order to support the development of regulations and policies that benefit all, sex, gender, and equity considerations must be integrated in research and policy from the outset. Moreover, we argue that the development of regulations must be in alignment with policy, and that progress on restricting exposure to tobacco SHS should be supported.

Legislation on tobacco smoking location restrictions in indoor public places and workplaces exists both in Canada and in many other countries. In Canada, the federal *Non-Smoker’s Health Act* restricts smoking and the use of ENDSs in workplaces and on public transportation. Further provincial, territorial, and municipal laws restrict smoking and ENDS use in public places. Smoking location restrictions have been associated with improved cardiovascular health outcomes, as well as reductions in mortality for smoking-related illnesses [3], preterm births, and hospital admissions for asthma [4]. In Canada, there is a federal commitment to apply gender-based analysis when developing and evaluating policies, programs, and initiatives. However, smoke-free policies have typically been gender-blind or applied a “one-size-fits-all” approach to reduce exposure to SHS [5,6]. Yet, there is clear evidence that sex-, gender-, and diversity- based factors impact tobacco use in general, and in particular, SHS exposure and its health effects [7]. For example, a systematic review revealed a significant association between combustible SHS and heart disease, chronic obstructive pulmonary disease (COPD), and stroke, with a greater risk for women than men for all three health outcomes [8]. Women and men tend to be exposed to SHS in different locations—men in the workplace and women in the home [9,10]. For these reasons, it is essential to apply a sex and gender lens to research on patterns of exposure and health effects to inform more nuanced policy and public health responses, especially as new products and substances are being regulated.

While there is currently less evidence on the health effects of exposure to secondhand cannabis smoke, cannabis smoke is similar in chemical composition to tobacco smoke (although in varying concentrations) [11]. A systematic review found that exposure to secondhand cannabis smoke results in the presence of cannabinoids in bodily fluids and psychoactive effects for those exposed [11]. There is evidence from some animal studies on the potential harms of secondhand cannabis smoke exposure. For example, a study with rats found that secondhand cannabis exposure produced negative cardiovascular health effects [12]. However, there is a lack of research on the long-term human health effects of secondhand cannabis smoke exposure [11], and no studies are available on the sex-specific effects of exposure.

As cannabis legalization is introduced in Canada (17 October 2018), it is expected that cannabis smoking will be included in tobacco smoking restriction by-laws. However, this will ultimately be the responsibility of individual provinces and territories [13]. As research on cannabis use patterns continues to emerge and policies and regulations regarding location restrictions of smoking cannabis are introduced, it is important that sex and gender differences and implications are investigated, analysed, and reported.

Compared to smoking tobacco, ENDSs and non-combustible forms of cannabis may be associated with lower relative harm both for users and for those who are exposed to the aerosol vapor. Currently, ENDSs are being investigated and debated regarding their potential as a harm reduction method, and as a tool to support smoking cessation [14]. Furthermore, the Lower Risk Cannabis Use Guidelines for Canada suggest people avoid using combustible cannabis, and use non-smoking methods such as vaping [15], as vaping natural cannabis has been associated with fewer respiratory effects compared to smoking cannabis [16].

In the current Canadian context of forthcoming legalization of cannabis and the regulation and expansion of the use of ENDS, further research is required to: (1) investigate the sex-specific health effects of involuntary exposure to ENDS and cannabis vaping products; (2) examine the health effects, including sex-specific effects, of exposure to new nicotine and cannabis vaping products and devices; and (3) examine how ENDS and cannabis vaping impact existing smoke-free legislation and patterns of involuntary exposure, all with particular attention to the implications for gender and health equity.

## 2. Health Effects of Involuntary Exposure to Vaping

Electronic nicotine delivery systems aerosolize a liquid that includes nicotine, a carrier ingredient (typically either propylene glycol or vegetable glycerine), and flavours, in varying concentrations [17]. There is evidence that exposure to aerosol vapour from ENDS may be less harmful than exposure to SHS [18]. Cigarette smoking is associated with higher airborne markers of nicotine [19]. However, non-smokers who are exposed to traditional cigarette smoke and e-cigarette vapour in the home demonstrate statistically similar levels of nicotine absorption [19]. A systematic review examining the health effects of passive exposure to e-cigarette vapour reported that while the health risk is not as pronounced as exposure to combustible cigarette smoke, bystanders may be exposed to a variety of harmful chemicals, including formaldehyde, heavy metals, and polycyclic aromatic hydrocarbons (PAHs) [18]. For example, measurements of air quality in indoor spaces following e-cigarette use have demonstrated an increase in ultrafine particles, including a 20% increase in carcinogenic PAHs [20]. A study in Greece reported an increase in the irritation of, and inflammatory markers of, the airways following 30 min of exposure to ENDS vapour [21]. Some authors have noted that the toxicants produced by ENDSs may be a greater risk for populations who are more vulnerable, including children and pregnant women [20]. Overall, there is a dearth of research examining the sex- or gender-specific health effects of exposure to ENDS vapour.

Cannabis vaping products are available that heat oil or liquid containing cannabis extracts or raw plant material to release aerosolized water vapour [22]. However, there is a lack of research examining the health effects of exposure to vapour from cannabis products, and no available studies examining sex-specific health effects. Reproductive aged and pregnant women have been identified as potentially vulnerable to the health effects of being exposed to toxicants from both ENDS and SHS from cannabis [23]. One study reported that health care providers are not asking pregnant women about their exposure to cannabis smoke or ENDS vapour [23]. Again, there is a dearth of evidence on sex and/or gender and the effects of exposure to ENDS and cannabis vapour.

More nuanced research is clearly needed on the health effects of involuntary exposure to ENDS and cannabis vaping. Meanwhile, however, products are rapidly evolving, while the harms associated with involuntary exposure to new products are largely unknown. There are ”heat not burn” (HNB) products that do not use an electronic heat source (e.g., Eclipse), that have been granted substantial equivalence status by the United States Food and Drug Administration, and are now approved for test marketing in the USA [24]. There are also HNB products such as IQOS [25], and higher nicotine containing ENDS products and brands such as Juul [26], which may be associated with greater health harms than standard nicotine vaping products. There is evidence that non-users who are exposed to e-cigarette products with higher nicotine content may absorb more nicotine [27]. The level of nicotine exposure may also be affected by the type of delivery device, the materials and battery used (specifically the voltage), and nicotine form (free-base or nicotine salts) [28]. Testing of the vapour released from a variety of commercially available e-cigarette products in the USA found that the majority of the nicotine was in the free-base form, and the measured nicotine concentration was higher than the labelled concentration [29]. In another study, among a group of non-smokers exposed to e-cigarette vapour, cotinine levels were higher following exposure to aerosol from tank-style e-cigarettes compared to disposable e-cigarettes [30].

Similar to e-cigarettes, cannabis vaping devices vary widely and the by-products and resulting health effects of exposure may differ depending on the carrier compounds, product materials, and heating capacity [22]. For example, along with cannabis smoking, Russell et al. identified “dabbing” as having the greatest potential for harm [16]. This route of administration involves the use of a modified water pipe in which a nail is heated with a blowtorch to vaporize cannabis concentrates (e.g., wax, shatter, budder). While the health effects of involuntary exposure to dabbing requires further investigation, there are numerous potential health risks for the user, including burns and explosions, and greater addiction due to the high potency of the concentrates used [16]. Again, further research is needed, particularly on the sex-specific health effects and gendered usage patterns, of both novel nicotine delivery products and cannabis vaping products.

## 3. Vaping in Smoke-Free Spaces

In Canada, restrictions on the use of e-cigarettes and vaping products tend to align with smoke-free by-laws. However, there is evidence that vaping products are commonly used in smoke-free locations [31], and that involuntary exposure to vaping products is likely to increase. E-cigarette use is common in smoke-free locations, particularly among young adults. In a US study, 74% of young adults used e-cigarettes in a smoke-free location [32]. Similarly, there is evidence that cannabis vaporizers are often used to “stealth vape” [31] in locations where smoking is prohibited (e.g., while at work) [13]. A study with older cannabis users in San Francisco found that some participants prefer using a vapour pen in public spaces because it is more discreet than smoking [33]. Similarly, a survey conducted with Canadians revealed that some people reported using e-cigarettes in locations where they were unable to smoke cigarettes [34].

Clearly, after legalization, the prevalence of cannabis vaping is likely to increase in Canada, similar to the experiences of US states that have legalized recreational cannabis [35], further exacerbating regulation and enforcement issues. Budney et al. have argued that the normalization of vaping nicotine and cannabis may increase frequency and misuse of cannabis among youth, particularly as the “positive features” of vaping will likely be used by the growing cannabis industry to encourage greater use and uptake [22]. Researchers have argued that implementing similar restrictions on cannabis smoking in public spaces is an important measure to prevent potential health harms and to support the gains made to denormalize smoking [11,36]. This concern is echoed in recommendations regarding the provincial regulation of cannabis in Canada. For example, the Health Officers Council of British Columbia recommend that the smoking and vaping of cannabis must align with tobacco smoking and nicotine vaping regulations, in order to avoid youth modelling [37].

However, it is important to avoid the errors of historical approaches to tobacco use and SHS exposure. Based on decades of research on tobacco exposure and policy impacts [5,38], it is very clear that patterns of use, involuntary exposure, and responses to policies are gendered. An Australian study found that women, young people, and people living on a low income are more often exposed to SHS in the home [9]. Similarly, a cross-European study reported that women were more often exposed in the home, while men and people with challenges in paying bills were more likely to be exposed to SHS in workplaces, restaurants, and bars [39]. It is to be expected that patterns of use and involuntary exposure to cannabis and vaping will also be gendered. Further research is needed on gendered patterns of use and sex-specific health effects of cannabis and nicotine vaping, to understand the implications of substituting ENDS and cannabis vaping for combustible tobacco and cannabis products, as well as the consequences of restricting vaping locations. For example, there could be a reduction in health-related harms for women and children living with combustible cigarette or cannabis smokers who substitute with/switch to ENDS or cannabis vaping. However, there is evidence that smoke-free legislation may not have equal benefits for all. Low-income women and men, and those who are renting a home (rather than owning) are more likely to be exposed to SHS, and may have limited capacity to reduce exposure to tobacco smoke in the home and workplace [40]. If there are significant health risks associated with indoor exposure to aerosol vapour, women and men who are experiencing social and economic disadvantage may be less likely to benefit from location restrictions on ENDS and nicotine vaping. Hence, comprehensive approaches to research that include sex-, gender-, and diversity-related factors that will inform equitable policies and regulations are urgently required. Investigation into both gender and SES differences in how vaping restrictions in public spaces impact involuntary exposure in private spaces are warranted to inform and improve policy responses to nicotine and cannabis vaping.

## 4. Conclusions

ENDS and cannabis vaping products pose significant challenges for researchers, decision-makers, and regulators, who must work quickly to keep pace with new products and a rapidly changing product, policy, and regulatory landscape such as Canada. To date, research on the health effects of involuntary exposure to ENDS and cannabis vaping products has been sex- and gender-blind, as was early tobacco research.

As new ENDS and cannabis vaping products continue to be introduced, and recreational cannabis is legalized in more jurisdictions, it is imperative that researchers and policy-makers reflect on the knowledge gained from tobacco policy implementation, smoking location restrictions, and denormalization policies, and consider sex- and gender-related factors in all research on the health effects of involuntary exposure to vaping products and cannabis policies. Specifically, researchers and policy-makers should explore how regulation and messaging efforts on cannabis smoking and vaping could reflect sex, gender, and equity concerns, and align with that of tobacco and nicotine vaping. This should be considered given overlapping issues such as location restrictions, SHS messaging, and related public health concerns. This way, the relevant science will be improved and the development of policy responses that are informed by sex, gender, and equity will be accelerated and enhanced.
